# Uptake of COVID-19 Vaccination Among Frontline Workers in California State Prisons

**DOI:** 10.1001/jamahealthforum.2022.0099

**Published:** 2022-03-11

**Authors:** Lea Prince, Elizabeth Long, David M. Studdert, David Leidner, Elizabeth T. Chin, Jason R. Andrews, Joshua A. Salomon, Jeremy D. Goldhaber-Fiebert

**Affiliations:** 1 Freeman Spogli Institute, Department of Health Policy, Stanford University School of Medicine, Stanford University, Stanford, California; 2 Stanford Law School, Stanford, California; 3 California Department of Corrections and Rehabilitation, Sacramento; 4 Department of Biomedical Data Science, Stanford University, Stanford, California; 5 Department of Medicine, Stanford University School of Medicine, Stanford, California

## Abstract

**Importance:**

Prisons and jails are high-risk environments for COVID-19. Vaccination levels among workers in many such settings remain markedly lower than those of residents and members of surrounding communities. The situation is troubling because prison staff are a key vector for COVID-19 transmission.

**Objective:**

To assess patterns and timing of staff vaccination in California state prisons and identify individual-level and community-level factors associated with remaining unvaccinated.

**Design, Setting, and Participants:**

This cohort study used data from December 22, 2020, through June 30, 2021, to quantify the fractions of staff and incarcerated residents who remained unvaccinated among 23 472 custody and 7617 health care staff who worked in roles requiring direct contact with residents at 33 of the 35 prisons operated by the California Department of Corrections and Rehabilitation. Multivariable probit regressions assessed demographic, community, and peer factors associated with staff vaccination uptake.

**Main Outcomes and Measures:**

Remaining unvaccinated throughout the study period.

**Results:**

Of 23 472 custody staff, 3751 (16%) were women, and 1454 (6%) were Asian/Pacific Islander individuals, 1571 (7%) Black individuals, 9008 (38%) Hispanic individuals, and 6666 (28%) White individuals. Of 7617 health care staff, 5434 (71%) were women, and 2148 (28%) were Asian/Pacific Islander individuals, 1201 (16%) Black individuals, 1409 (18%) Hispanic individuals, and 1771 (23%) White individuals. A total of 6103 custody staff (26%) and 3961 health care staff (52%) received 1 or more doses of a COVID-19 vaccine during the first 2 months vaccines were offered, but vaccination rates stagnated thereafter. By June 30, 2021, 14 317 custody staff (61%) and 2819 health care staff (37%) remained unvaccinated. In adjusted analyses, remaining unvaccinated was positively associated with younger age (custody staff: age, 18-29 years vs ≥60 years, 75% [95% CI, 73%-76%] vs 45% [95% CI, 42%-48%]; health care staff: 52% [95% CI, 48%-56%] vs 29% [95% CI, 27%-32%]), prior COVID-19 infection (custody staff: 67% [95% CI, 66%-68%] vs 59% [95% CI, 59%-60%]; health care staff: 44% [95% CI, 42%-47%] vs 36% [95% CI, 36%-36%]), residing in a community with relatively low rates of vaccination (custody staff: 75th vs 25th percentile:, 63% [95% CI, 62%-63%] vs 60% [95% CI, 59%-60%]; health care staff: 40% [95% CI, 39%-41%] vs 34% [95% CI, 33%-35%]), and sharing shifts with coworkers who had relatively low rates of vaccination (custody staff: 75th vs 25th percentile, 64% [95% CI, 62%-66%] vs 59% [95% CI, 57%-61%]; health care staff: 38% [95% CI, 36%-41%] vs 35% [95% CI, 31%-39%]).

**Conclusions and Relevance:**

This cohort study of California state prison custody and health care staff found that vaccination uptake plateaued at levels that posed ongoing risks of further outbreaks in the prisons and continuing transmission from prisons to surrounding communities. Prison staff decisions to forgo vaccination appear to be multifactorial, and vaccine mandates may be necessary to achieve adequate levels of immunity in this high-risk setting.

## Key Points

**Question:**

In California prisons, what proportion of prison staff who have direct contact with residents are unvaccinated, and what are their characteristics?

**Findings:**

In this cohort study of 23 472 custody staff and 7617 health care staff in California state prisons, 14 317 custody staff (61%) and 2819 (36%) health care staff remained unvaccinated through June 30, 2021, despite widespread vaccine availability. Unvaccinated staff were younger and more likely to have had COVID-19; they were also more likely to work alongside other unvaccinated staff and live in communities with relatively low rates of vaccination.

**Meaning:**

The study results suggest that low vaccination rates among prison staff pose continuing risks.

## Teaser

This cohort study examines rates of COVID-19 vaccination among custody staff and health care staff in California state prisons.

## Introduction

Prisons and jails are particularly high-risk environments for COVID-19. Since the pandemic began, there have been more than 550 000 COVID-19 cases among residents and staff in carceral settings in the US and nearly 3000 deaths, and infection rates among residents are 4 to 5 times higher than those in the general community.^1,2,3,4,5^ In California state prisons, approximately 1 in 4 residents has had COVID-19, and 241 have died.^6,7^ Correctional staff who socially mix in the communities where they live and also have direct contact with prison residents are likely to be a significant source of introduction of SARS-CoV-2 infection into prisons.^4,8^

Beginning in December 2020, California prioritized prison residents and employees for receiving COVID-19 vaccination. By mid-October 2021, 23% of California’s inmates remained unvaccinated, whereas almost 40% of all of California’s prison staff remained unvaccinated.^9^ Nationally, COVID-19 vaccine coverage rates among prison staff have been lower than rates in the wider community. To redress this situation, the federal government and several states, including California, have attempted to implement mandates, prompting staff resignations and lawsuits that have forestalled implementation of these requirements.^10,11,12^

Vaccine hesitancy among workers in high-risk settings, like prisons, is not well understood. We analyzed the uptake of COVID-19 vaccination among more than 31 000 staff whose jobs put them in direct contact with California’s state prison residents. The goal was to describe patterns of vaccination in this population and identify individual and workplace characteristics associated with low vaccine uptake.

## Methods

### Data

The California Department of Corrections and Rehabilitation (CDCR) provided anonymized person-day level data for this cohort study. The data included comprehensive information on CDCR-performed or self-reported polymerase chain reaction and antigen testing and vaccination for prison staff and residents (January 1, 2020, through June 30, 2021), plus information on incident cases among residents through September 25, 2021. The staff data also included information on demographic characteristics, zip code of residence, and shifts that staff members worked at which prison. Statistics on SARS-CoV-2 infection by zip codes came from California Department of Public Health data. The institutional review board of Stanford University approved the study, and informed consent was waived because of the use of deidentified data. This study followed the Strengthening the Reporting of Observational Studies in Epidemiology (STROBE) reporting guidelines for cohort studies.

### Study Sample

The study focused on correctional staff who worked in CDCR prisons during a 191-day period from December 22, 2020, when vaccination was first offered to staff, through June 30, 2021. The study period ended before proposals for and debate regarding mandatory vaccination for prison staff flared in California and before the delta and omicron variant surges (eAppendix in the Supplement). We restricted the analytic sample to staff who worked 5 or more shifts between April 1, 2021 (by which time vaccination was available for any staff member who wanted to receive it) and June 30, 2021, were employed with a designation of custody or health care (excluding contract employees), and worked in roles that involved direct contact with residents (direct care). Two of CDCR’s 35 prisons were excluded because of missing or incomplete staff data. Staff with missing values for variables of interest (<1% of those who met the previously described eligibility criteria) were excluded.

### Variables

The outcome of interest in the primary analyses was an indicator variable that denoted staff who had not been vaccinated by the end of the study period. We also analyzed the subgroup of staff who had neither been vaccinated nor had a history of SARS-CoV-2 infection.

Two variables allowed us to focus the analyses on direct care staff. First, CDCR’s main position designation identified 2 large groupings of staff whose positions typically involve face-to-face contact with residents: custody and health care staff. Second, a direct care variable permitted further narrowing to staff with regular direct contact with residents. These inclusion criteria were developed through extensive consultation with CDCR officials.

Being unvaccinated was defined as having received no doses of a COVID-19 vaccine. No history of SARS-CoV-2 infection was defined as having no confirmed positive result from a polymerase chain reaction or antigen test before June 30, 2021. Notably, 402 vaccinated staff (3%) in the study sample had a first positive test result after vaccination. The CDCR’s staff testing program has involved a combination of mandatory and voluntary testing; most of the testing has been routinely performed by CDCR itself, although CDCR employees are required to provide information about any positive test results, and self-reported results were included in the data. We were unable to identify self-report tests with confidence because of incomplete or miscoded data. Based on conversations with CDCR staff, the number of outside test results in the data was very small, especially during the study period. The CDCR’s resident testing program comprises routine risk-based and surveillance testing and testing in response to detected outbreaks.^13,14^

The demographic information on staff members included age group, self-reported racial or ethnic group, sex, recorded zip code of residence, and whether the individual had any history of SARS-CoV-2 infection. To explore the association between staff members’ vaccination status and 2 potential environmental factors, lack of vaccination uptake in communities where they lived and among fellow workers, we created 2 additional variables. First, we used the California Department of Public Health area-level data to calculate the cumulative fraction of unvaccinated individuals aged 20 to 64 years old on June 1, 2021, in the most recent zip code in which each staff member resided (see eTable 1 in the Supplement for counts of included zip codes by prison). Second, we calculated the proportion of unvaccinated coworkers who worked in the same job classification (custody or health care) on the same shift, day, and prison as the staff member being analyzed. This variable was a composite, cross-sectional measure created by assigning a weight to each staff person’s coworkers based on the total number of shifts worked together during the study period while either vaccinated (received ≥1 dose of vaccine) or unvaccinated (eAppendix in the Supplement).

### Statistical Analysis

We plotted vaccination uptake and detected prior infection among custody and health care staff (separately) during the study period. Next, for each prison, we compared the proportions of staff and residents who remained unvaccinated at the end of the study period. We repeated this approach, comparing proportions and examining cumulative risk, after redefining the vulnerable groups of interest to include staff and residents, respectively, who were neither vaccinated nor had a history of prior detected infection by the end of the study period.

Finally, to identify characteristics of staff members who remained unvaccinated, we fit staff-level multivariable probit regression models (1 each for custody and health care staff). The outcome variable in these models was remaining unvaccinated at the end of the study period. The control variables included race and ethnicity, age group, sex, detected infection at any time before vaccination (including before the study period), and the variables constructed to measure levels of nonvaccination in each staff member’s residential neighborhood and shift cohort, as described previously. We also included an indicator of the most-worked shift (night, day, or swing), the number of shifts worked during the study period, and the average number of shifts worked per week, as well as a prison fixed effect, indicating the prison where a staff member worked most.

Results from the multivariable analyses are reported as the predicted probabilities that staff members in the groups of interest remained unvaccinated through June 30, 2021. These predicted probabilities indicate the average predicted values for all cases obtained by holding 1 variable at a fixed value and all other independent variables at their observed values. For the continuous variables that measured the proportion of unvaccinated individuals in residential zip codes and the shift cohorts, we report predicted values at the 25th, 50th, and 75th percentile of the distributions for custody and health care staff, respectively. All analyses were performed using Stata, version 16.1 (StataCorp).

## Results

### Prevalence of Vaccination and Prior Infection

**Figure 1.**
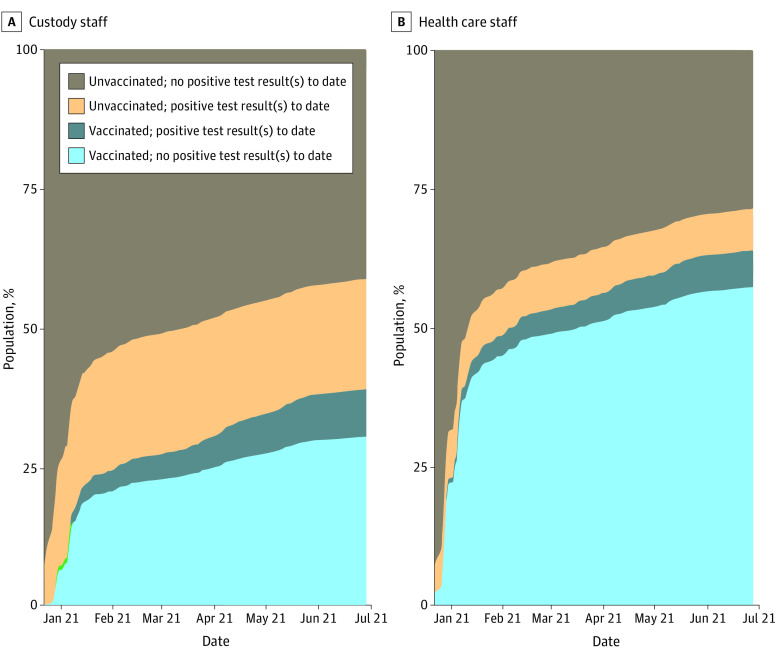
Cumulative Vaccination and COVID-19 Infection for Custody and Health Care Staff Cumulative counts among custody and health care direct care staff who had active shifts in June 2021 (n = 23 472 for custody and n = 7617 for health care staff). Vaccinated indicates receipt of 1 or more doses of any COVID-19 vaccine, and not vaccinated indicates receipt of 0 doses of vaccine as of a given date. No postive test result indicates no positive result for a recorded test for SARS-CoV-2 infection as of a given date. Positive test result indicates at least 1 recorded positive test result or infection as of a given date. Counts do not reflect the order of vaccination uptake and SARS-CoV-2 infection; rather, they shifted proportionately as staff members moved from category to category.

Uptake of COVID-19 vaccination among staff was brisk initially but plateaued within a couple of months. Figure 1 shows that from December 22, 2020, through February 15, 2021, coverage with at least 1 vaccine dose increased from 0% to 26% for custody staff and 0% to 52% for health care staff. Thereafter, the pace of vaccination coverage slowed, reaching 39% for custody staff and 64% for health care staff by June 30, 2021. Figure 1 also shows that the surge in COVID-19 cases during the winter of 2020 to 2021 was associated with substantial increases in the cumulative prevalence of confirmed COVID-19 cases among staff. Through December 22, 2020, CDCR had previously detected COVID-19 in 5% of the custody staff in the study sample and 2% of health care staff; by June 30, 2021, these proportions had increased to 27% and 12%, respectively. A total of 16 789 unvaccinated custody staff (72%) and 6550 unvaccinated health care staff (88%) had no recorded history of SARS-CoV-2 infection (eFigures 1 and 2 in the Supplement reproduce the information shown in Figure 1 for each prison).

**Figure 2.**
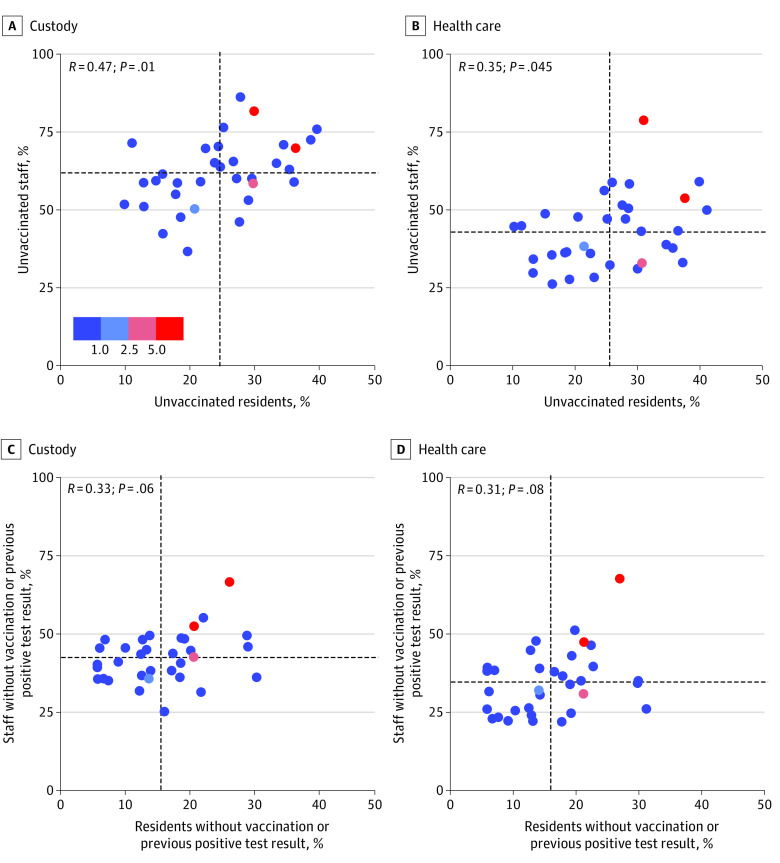
Percentage of Custody Staff, Health Care Staff, and Residents of California State Prisons Who Were Unvaccinated and Did Not Previously Test Positive for SARS-CoV-2 by Prison on June 30, 2021, and Cumulative Incidence of Infection Among Residents From July 1, 2021, Through September 25, 2021 Proportions of unvaccinated custody and health care staff and residents (A and B) and proportions of unvaccinated custody and health care staff and residents who also had no previous positive test result (C and D) as of June 30, 2021, at each of 33 California Department of Corrections and Rehabilitation prisons. Cumulative incidence for residents was based on total incidence of SARS-CoV-2 infection between July 1, 2021, and September 25, 2021. Vaccinated indicates receipt of 1 or more doses of any COVID-19 vaccine, and not vaccinated indicates receipt of 0 doses of vaccine. Each point on the graph represents an individual prison. The horizontal and vertical dashed lines represent the institutional mean. The color key is in percentage points.

Thus, 14 369 custody staff (61%) and 2822 health care staff (37%) remained unvaccinated at the end of the study period. These proportions varied widely across the 33 prisons in the sample, from 37% to 86% among custody staff and 26% to 79% among health care staff. Figure 2A and B plot these proportions against proportions of unvaccinated residents (eTable 2 in the Supplement). Coverage among custody staff was lower than coverage among residents in every prison; this decrement ranged from 17 to 60 percentage points. Coverage among health care staff was lower than coverage among residents in all but 1 prison, with decrements ranging from to 1 to 48 percentage points. Prisons that had higher proportions of unvaccinated custody staff also tended to have higher proportions of unvaccinated health care staff (Spearman ρ, 0.57; *P* = .01).

The cumulative proportion of residents at each prison who became infected during the 3 months following the study period ranged from 0% to 7.3% (mean, 0.8%; 6 prisons greater than the mean) (Figure 2; eTable 2 in the Supplement). The fraction of unvaccinated custody staff was more strongly correlated with subsequent resident infection rates than the fraction of unvaccinated health care staff or unvaccinated residents (Spearman ρ for custody staff, 0.35; *P* = .05; for health care staff, 0.22; *P* = .23; for residents, 0.26; *P* = .15). Of the 6 prisons with the largest proportions of residents infected during this 3-month period, 4 had greater than average levels of unvaccinated custody staff, 4 had greater than average levels of unvaccinated health care staff, and 5 had greater than average levels of unvaccinated residents. Patterns remained consistent when we refined the definition of the vulnerable population to include only individuals who remained unvaccinated and with no history of COVID-19 (Figure 2C and D).

### Correctional Staff Risk Factors for Remaining Unvaccinated

The study sample consisted of 31 089 prison staff who were employed at 33 prisons; 23 472 (75.5%) were custody staff and 7617 (24.5%) were health care staff (Table). Most were aged 30 to 49 years. Most custody staff were male (19 721 [84%]), and most health care staff were female (5434 [71%]). Two-thirds of custody staff were Hispanic (9008 [38%]) or White (6666 [28%]), while half of health care staff were Asian (2148 [28%]) or White (1771 [23%]).

**Table.** Individual and Shift Characteristics of the Study Sample of Custody and Health Care Direct Care Staff at 33 California State Prisons

Characteristic	No. (%)^a^	
	Custody staff (n = 23 472)	Health care staff (n = 7617)
Age category, y		
18-29	2862 (12)	422 (6)
30-39	7724 (33)	2076 (27)
40-49	7765 (33)	2362 (31)
50-59	4310 (18)	1886 (25)
≥60	811 (3)	871 (11)
Race and ethnicity		
Asian/Pacific Islander	1454 (6)	2148 (28)
Black	1571 (7)	1201 (16)
Hispanic	9008 (38)	1409 (18)
White	6666 (28)	1771 (23)
Other/unknown^b^	4773 (20)	1088 (14)
Sex		
Women	3751 (16)	5434 (71)
Men	19 721 (84)	2183 (29)
No history of COVID-19 infection before June 30, 2021^c^	17 052 (73)	6692 (88)
Remained unvaccinated through June 30, 2021	14 369 (61)	2822 (37)
Fraction of individuals aged 20-60 y vaccinated in zip code of residence, mean (SD)^d^	56 (12)	52 (13)
Institution/work shift characteristics		
Fraction of shift coworkers not vaccinated, mean (SD)^e^	60 (11)	34 (10)
Most worked shift		
Night	3170 (14)	699 (9)
Day	13 510 (58)	5014 (66)
Swing	6792 (29)	1904 (25)
No. of shifts during study period, mean (SD)^f^	126 (28)	123 (45)
No. of shifts/week, mean (SD)^f^	4.7 (0.84)	4.6 (1.32)

Abbreviations: CDCR, California Department of Corrections and Rehabilitation; CDPH, California Department of Public Health.

^a^ The analytic data set includes 31 089 custody or health care staff who worked at least 5 direct care shifts between April, May, and June 2021 and at least 1 direct care shift in each of those months in any 1 of 33 CDCR prisons.

^b^ The other/unknown category of race includes American Indian or Alaska Native (approximately 0.4% of total sample) and other (approximately 1% of total) and unknown or missing (approximately 1.7% of total).

^c^ No history of COVID-19 infection indicates no positive result on any polymerase chain reaction or antigen diagnostic test for SARS-CoV-2 infection before the first dose of vaccination, or before the end of the study period for unvaccinated individuals.

^d^ Vaccination rates of zip code of residence are based on cumulative CDPH vaccination rates as of June 1, 2021, in the zip code where the staff person lived on the most recently recorded work shift.

^e^ Fraction of coworkers not vaccinated is a count of all coworkers by date, shift, and prison and is weighted by the number of shifts worked with a given coworker.

^f^ Number of shifts worked and number of shifts per week are based on shifts worked between December 22, 2020, and June 30, 2021 (for the latter, weeks in which there were 0 shifts worked were excluded in calculation).

Among custody staff, 14 369 (61%) were unvaccinated (ie, had received no doses of vaccine) and 72% had no confirmed COVID-19 by the end of June 2021. Among health care staff, 2882 (37%) were unvaccinated and 88% had no confirmed infection. Custody staff resided in zip codes with a slightly higher rate of unvaccinated adults than health care staff (56% vs 52%; *P* < .001) and had a much higher fraction of unvaccinated coworkers in their work cohorts (60% vs 34%; *P* < .001). The 2 types of workers had similar shift patterns.

**Figure 3.**
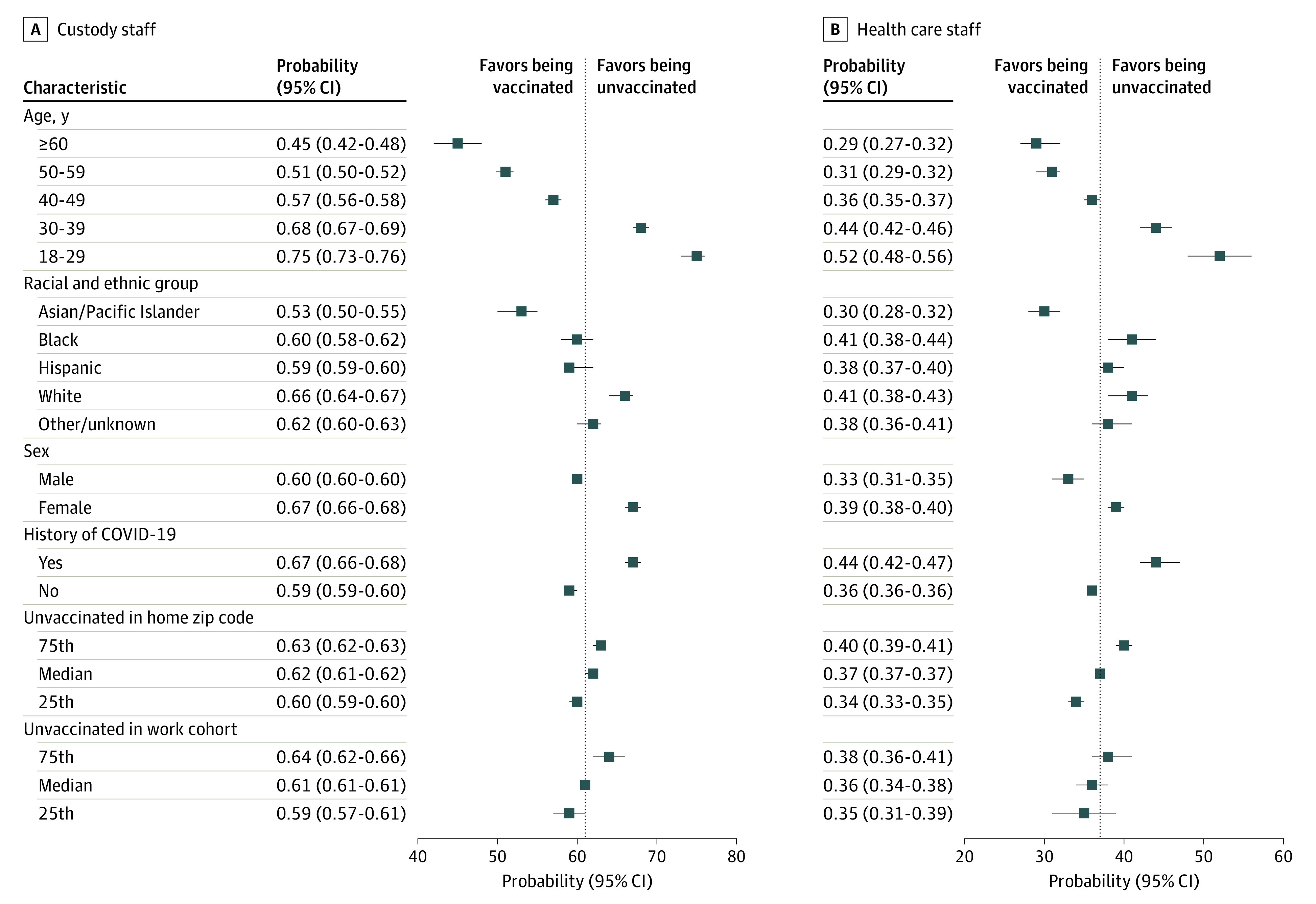
Predicted Probabilities of Remaining Unvaccinated Through June 30, 2021, for Custody and Health Care Staff From the Multivariable Regression Models A and B, Predicted probability (average adjusted margins) of being unvaccinated by June 30, 2021, for custody staff (n = 23 472) and health care staff (n = 7617), respectively, which was estimated using a multivariable probit model with robust standard errors, clustering on the main prison of employment. History of COVID-19 indicates a positive result on any polymerase chain reaction or antigen diagnostic test for SARS-CoV-2 infection during the study period. Vaccination rates of zip code of residence are based on cumulative California Department of Public Health vaccination rates as of June 1, 2021, in the zip code where the staff person lived on the most recent work shift. Fraction of coworkers not vaccinated is a count of all coworkers by date, shift, and prison and was weighted by the number of shifts worked with a given coworker. The model controlled for all variables as well as an indicator of the shift (night, day, or swing) most often worked, the number of shifts worked during the study period, the average number of shifts worked on active weeks, and prison fixed effects, representing the main prison in which a staff member worked during the study period. For unvaccinated individuals in a home zip code and unvaccinated individuals in a work cohort, which were continuous variables in the model, we predicted at the 25th, 50th, and 75th percentiles (49%, 57%, and 63% for the former and 53%, 59%, and 67% for the latter for custody staff; 44%, 53%, and 61% for the former and 26%, 31%, and 39% for the latter for health care staff).

Adjusted analyses showed a strong age gradient to vaccination uptake (Figure 3; eTables 3 and 4 in the Supplement). For example, custody staff aged 18 to 29 years were 30 percentage points more likely to remain unvaccinated than their colleagues 60 years or older (predicted probability 0.75; 95% CI, 0.73-0.76 for age 18-29 years; 0.45; 95% CI, 0.42-0.48 for 60 years or older), while health care staff aged 18 to 29 years were 23 percentage points more likely to remain unvaccinated than their colleagues 60 years or older (predicted probability, 0.52; 95% CI, 0.48- 0.56 for age 18-29 years; 0.29; 95% CI, 0.27-0.32 for 60 years or older). Custody and health care staff with a history of COVID-19 were 8 percentage points more likely to remain unvaccinated than their colleagues without such a history (predicted probability for history/no history: custody staff: 0.67 [95% CI, 0.66-0.68] vs 0.59 [95% CI, 0.59-0.60; health care staff: 0.44 [95% CI, 0.42-0.47] vs 0.36 [95% CI, 0.36-0.36]).

Residing in zip codes with relatively large proportions of unvaccinated residents aged 20 to 64 years was associated with slightly higher probability of remaining unvaccinated: 3 percentage points higher for custody staff, comparing the 75th percentile with the 25th percentile, and 6 percentage points higher for health care staff (both differences significant; predicted probability for 75th/25th percentile: custody staff: 0.63 [95% CI, 0.62-0.63; *P* < .001] vs 0.60 [95% CI, 0.59-0.60; *P* < .001]; health care staff: 0.40 [95% CI, 0.39-0.41; *P* < .001] vs 0.34 [95% CI, 0.33-0.35; *P* < .001]). Similarly, staff members who tended to share shifts with other staff who had lower levels of vaccination had a higher probability of remaining unvaccinated: 5 percentage points higher for custody staff, comparing the 75th percentile with the 25th percentile, and 3 percentage points higher for health care staff (custody staff difference was significant, whereas health care staff difference was not; predicted probability for 75th/25th percentile: custody staff: 0.64 [95% CI, 0.62-0.66; *P* = .02] vs 0.59 [95% CI, 0.57-0.61; *P* = .02]; health care staff: 0.38 [95% CI, 0.36-0.41; *P* = .41] vs 0.35 [95% CI, 0.31-0.39; *P* = .41]). See the eAppendix in the Supplement for discussion of sensitivity analyses and presentation of results.

## Discussion

This cohort study of California state prison custody and health care staff found that staff vaccine coverage lagged far behind vaccine coverage among residents for the first half of 2021. Some prisons had relatively low rates of coverage for staff and residents, creating substantial risks of ongoing outbreaks. These results highlight the multifactorial nature of reasons for hesitancy, which are a function of demographic factors, community environment, peer behaviors, and other considerations that this study was not designed to measure.

Personal decisions regarding vaccination are complex.^15^ Among prison staff in California, we detected evidence of multifactorial explanations. Lower uptake among younger staff and those with a history of COVID-19 were consistent with findings from other settings and suggest perceptions of one’s personal risk of infection or serious health consequences affect decision-making.^16^ The positive association we identified between low uptake and working with unvaccinated staff and living in communities with low vaccination levels is not surprising. People can be swayed by those around them and seek out those whose beliefs align with their own.^17,18^ However, the risk such staff pose to residents is likely to be especially high, as they are at relatively high risk of acquiring SARS-CoV-2 infection and bringing it into the workplace. While one might expect that in high transmission risk settings like prisons staff would seek to protect themselves, especially if those around them are less well protected via vaccination, the findings of this study highlight the potential interplay of social dimensions and individual factors in the decision to obtain COVID-19 vaccination.

To address the risks posed by unvaccinated workers to vulnerable institutionalized populations, vaccine mandates have been enacted or proposed.^19^ Although prison worker unions in these settings generally endorse vaccination, they oppose mandates and have fought them on picket lines and in court. In California, vaccine mandates for state prison employees have come from the executive branch^6^ and the courts.^20^ However, both have been challenged in federal court, and neither is currently in force.^21^

This study’s findings that correctional staff are more likely to remain unvaccinated if the people around them are unvaccinated suggests that important peer effects may be lessening vaccination uptake. Delivery of staff vaccination in the context of prisons, with or without a mandate, should be designed with peer effects in mind. For example, ensuring that individual staff members have access to vaccination in venues and at times when they can avoid being observed by their coworkers may be important. Likewise, as vaccination coverage increases in the prison and in particular staff groups (eg, custody staff, night shift), communicating the current status of the cohort as a whole may help to shift a staff member’s perception of their peers’ actions.

### Limitations

This study has several limitations. First, its generalizability to prisons outside California and workers in other high-risk settings is unknown. Second, designing and implementing optimal strategies for boosting uptake of COVID-19 vaccination requires detailed understanding of the knowledge, beliefs, and preferences of those who are hesitant, and we did not measure these factors. Similarly, the measures of the effects of coworkers and home communities are crude; studies with qualitative and mixed-method designs are better able to engage with the nuances of personal motivations. Third, CDCR records may have missed vaccinations obtained by some staff outside CDCR’s program. However, such misclassification is likely to have been uncommon because CDCR required notification, and staff who did not show proof of vaccination were required to undergo a continuous testing regimen. Fourth, the adjusted analyses controlled for the level effects of institutions under the assumption that the levels are separate and independent. By doing so we were unable to examine how institution-wide practices and internal shocks affect vaccine decisions. For example, there is some indication that institutions with more staff deaths have higher vaccination rates (eFigure 3 in the Supplement). Future analyses could incorporate a more flexible model that would allow the exploration of institution-level factors. Finally, because of the rapid rollout and sharp uptake of vaccination among staff members who chose to get vaccinated, we were unable to analyze how changes over time in coworker vaccination rates were directly associated with vaccination uptake.

## Conclusions

This cohort study found that despite ongoing risks of COVID-19 outbreaks in high-risk carceral settings, like California’s state prisons, vaccination rates among prison staff continue to lag behind those of incarcerated residents. Younger staff and staff who have had COVID-19 may be disproportionately unlikely to receive vaccinations because they perceive themselves to be at low risk of infection or disease compared with their older colleagues or those who have not had a prior infection. However, social considerations at home and work also appear important, leading to some prisons and work cohorts with persistently lower than average levels of staff vaccination. While effective and acceptable approaches to rapidly increasing vaccination coverage among correctional staff remain elusive, failing to develop and implement them prolongs health risks to staff themselves and the communities in which they reside, as well as to incarcerated residents.

With almost 1.2 million people currently residing in state and federal prisons in the US,^22^ addressing the risk of COVID-19 transmission and subsequent outbreaks, especially from highly transmissible viral variants, is a critical public health priority. Despite substantial levels of natural and acquired immunity in many incarcerated populations, COVID-19 risks continue to loom large.^23,24^

## Notes

Supplement.
eAppendix.
**eTable 1.** Counts of direct care staff, different zip codes of residence, and different counties of residence among CDCR custody and healthcare staff **eTable 2.** Fraction unvaccinated and fraction not vaccinated and without history of Covid-19) for prison for staff and incarcerated people; Cumulative incidence following the study period **eTable 3. **Predicted probabilities and confidence intervals for adjusted analysis **eTable 4. **Coefficients from multivariable analyses **eTable 5. **Sensitivity analyses results matrix **eFigure 1. **Variation among prisons in vaccine and test-positive scale-up over time, Custody staff **eFigure 2. **Variation among prisons in vaccine and test-positive scale-up over time Healthcare staff **eFigure 3. **Counts of COVID-19 deaths among staff and fraction of direct care staff vaccinated, by institution (December 2020 – June 30, 2021)
