# Supplementary material for: Uptake of COVID-19 Vaccination Among Frontline Workers in California State Prisons
Source: JAMA Health Forum. 2022 Mar 11;3(3):e220099. doi: 10.1001/jamahealthforum.2022.0099 (PMC8917424; doi:10.1001/jamahealthforum.2022.0099)
Supplement: Supplement. — eAppendix. eTable 1. Counts of direct care staff, different zip codes of residence, and different counties of residence among CDCR custody and healthcare staff eTable 2. Fraction unvaccinated and fraction not vaccinated and without history of Covid-19) for prison for staff and incarcerated people; Cumulative incidence following the study period eTable 3. Predicted probabilities and confidence intervals for adjusted analysis eTable 4. Coefficients from multivariable analyses eTable 5. Sensitivity analyses results matrix eFigure 1. Variation among prisons in vaccine and test-positive scale-up over time, Custody staff eFigure 2. Variation among prisons in vaccine and test-positive scale-up over time Healthcare staff eFigure 3. Counts of COVID-19 deaths among staff and fraction of direct care staff vaccinated, by institution (December 2020 – June 30, 2021) [file jamahealthforum-e220099-s001.pdf]

Supplemental Online Content

Prince L, Long E, Studdert DM, et al. Uptake of COVID-19 vaccination among frontline workers in California state prisons. *JAMA Health Forum*. 2022;3(3):e220099. doi:10.1001/jamahealthforum.2022.0099

eAppendix.

eTable 1. Counts of direct care staff, different zip codes of residence, and different counties of residence among CDCR custody and healthcare staff

eTable 2. Fraction unvaccinated and fraction not vaccinated and without history of Covid-19) for prison for staff and incarcerated people; Cumulative incidence following the study period

eTable 3. Predicted probabilities and confidence intervals for adjusted analysis

eTable 4. Coefficients from multivariable analyses

eTable 5. Sensitivity analyses results matrix

eFigure 1. Variation among prisons in vaccine and test-positive scale-up over time, Custody staff

eFigure 2. Variation among prisons in vaccine and test-positive scale-up over time Healthcare staff

eFigure 3. Counts of COVID-19 deaths among staff and fraction of direct care staff vaccinated, by institution (December 2020 – June 30, 2021)

This supplemental material has been provided by the authors to give readers additional information about their work.

© 2022 Prince L et al. *JAMA Health Forum.*

Analytic cohort design

Study period

The first day of the study period was the first day that vaccines were offered and recorded in the CDCR dataset, December 22, 2020. The study period goes through June 30, 2021. We chose to analyze data through June 30, 2021 for several reasons. First, we wanted to examine how vaccine uptake is correlated with subsequent outbreaks at prisons (Figure 2 in main text). Second, we are interested in mutable factors which influence vaccine uptake. After June 2021, the Delta variant became prevalent in CDCR prisons and talk of mandated vaccines for corrections workers began. Because it would be difficult to separate the difference in characteristics of late adopters from the influence of these two important changes, we chose to cut off our study period prior to July 1, 2021.

Dataset

Daily data extracts provided by the California Department of Corrections and Rehabilitation (CDCR) included unique pseudo-identifiers for both staff and incarcerated residents enabling us to follow them over time and included comprehensive information on PCR and antigen testing and vaccination. Our sample included custody and healthcare staff who worked in a CDCR prison between January 1, 2020 through June 30, 2021. The staff data were provided per day per shift, so any staff person who worked an active shift on a given day had an observation. We did not have information about leaves of absence, time off, resignation or termination other than what was imputed based on the active shift-days.

To limit our analytic dataset to correctional staff with the greatest likelihood of direct contact with incarcerated residents, we imposed several inclusion/exclusion criteria. Our analytic cohort was drawn from staff in 33 of CDCRs 35 prisons. Two of CDCR's 35 prisons were excluded from the analysis due to missing or incomplete staff data at those prisons at the time of analysis. We included only staff designated as custody or healthcare workers in one of the 33 included prisons (~63% of total CDCR staff). Other possible designations are contractor, education, and operations. Among the healthcare and custody staff, we included people designated as "direct-care" staff, meaning that they were classified by CDCR as having regular in-person contact with residents (~98% of custody and healthcare staff). We excluded staff who were missing data for any of our covariates (<1%), with the exception of missing race, which was coded under "Other/Unknown". We excluded staff who did not work at least five shifts between April 2021 and June 2021 (the period after vaccination was available to any staff person; 275 healthcare and 181 custody staff were excluded). Some prison staff (638 in our study sample) worked across multiple prisons, they entered the analysis assigned to their main institution. There were 3 staff who were appointed as custody and healthcare at different times. These were excluded from our analysis. There were 171 staff who had a record of being vaccinated in the community before their first recorded shift at CDCR. These were excluded from the analysis. Importantly, the fraction of co-workers vaccinated variable was constructed based on the entire custody or healthcare direct-care staff, prior to exclusions.

Outcome

Remained unvaccinated: CDCR staff were offered vaccines at their place of work beginning on December 22, 2020. Vaccine uptake was voluntary through the end of our study period. All vaccines administered on-site are recorded in CDCR data (and reflected in our dataset), and vaccines administered in the community were obtained from the California Immunization Registry, provided staff members gave their consent. The outcome reflects that the staff member remained unvaccinated (no doses of vaccine administered) through June 30, 2021.

Variables

Age category: We collapsed age into categories: 18-29, 30-39, 40-49, 50-59, 60+. These categories are consistent with or finer than those available in CDC and CDPH datasets (See, https://covid19.ca.gov/state-dashboard/#ethnicity-gender-age or https://covid.cdc.gov/covid-data-tracker/#demographicsovertime, for example).

Race group. We grouped race by race and ethnicity information provided by CDCR. Race categories and their components are as follows: Asian/PI (Asian or Filipino or Pacific Islander); Black (Black); Hispanic (Hispanic + Cuban + Mexican); White (White); Other/Unknown (includes American Indian or Alaskan Native (~0.4% of total sample) and other (~1% of total) and unknown or missing (~17% of total).

Gender: Gender is based on self-report of gender.

History of Covid-19: The staff data included comprehensive information on PCR and antigen testing for SARS-CoV-2 infection, with the first recorded test on March 18, 2020. Testing was voluntary and/or mandatory and occurred at varying frequency over time. Because testing was infrequent through early 2020 (see counts below), we may underestimate the proportion of staff with any history of Covid-19. Despite this, we feel confident in our estimate of fraction of staff without any positive test at the end of June 2021 because of ramped up testing starting in mid-2020 (when non-fully-vaccinated staff have been tested twice-weekly and any staff who

© 2022 Prince L et al. *JAMA Health Forum.*

report symptoms or work in a location with an outbreak have also been tested) in combination of the timing of most community and prison outbreaks from mid-2020 and onward.

Monthly counts of SARS-CoV2 tests and test-positive for direct care custody and health staff

Month	Tests Administered	Tests Positive
**2020**		
March	13	12
April	33	31
May	429	66
June	9,729	215
July	66,684	831
August	55,047	535
September	72,164	290
October	81,580	382
November	97,484	1,163
December	121,075	3,272
**2021**		
January	109,535	1,400
February	102,838	356
March	124,159	293
April	128,069	230
May	87,398	169
June	93,663	205

*Counts are for the 33 prisons included in the analytic sample.

Unvaccinated in zip code: Fraction of unvaccinated adults in staff home-zip code is based on the cumulative percentage of the 20-64 year-old population in a given zip-code who received 1+ doses of vaccination by June 1, 2020. Data are from CDPH (see reference in main text). We used the last known zip code of staff members to create this measure.

Unvaccinated in work cohort: To build a cross-sectional measure of peer vaccine take-up, we counted, for each staff-shift, all co-workers on each prison-shift-day and all coworkers who were unvaccinated on each prison-shift-day. The individual-level measure was $\frac{\sum_{S=1}^{N} Us-1}{\sum_{S=1}^{N} Ts-1}$ where *N* is the total number of shifts worked by an individual staff person during the study period, *Us* is the number of unvaccinated workers on a particular prison-shift-day and *Ts* is the total number of workers on a particular prison-shift-day. Note, we subtract the individual for whom the measure is calculated in the counts of unvaccinated and of total workers to avoid a reflection issue.

See Appendix Table 5 for a sensitivity analysis limiting the analysis to prisons with the greatest within-institution inter-quartile range (as a measure of spread).

Shift variables: We created a categorical variable denoting which shift a staff person worked most often (based on raw count of shifts worked over the study period): day, night, or swing shift. We controlled for the total number of shifts worked during the study period, the mean shifts per week worked (weeks with zero shifts were not counted in the mean), and their interaction.

Prison Fixed effects: We included prison-level fixed effects to control for stable heterogeneity between prisons.

IRB and ethics approval

The study was approved by the institutional review board (IRB) at Stanford University (protocol numbers IRB-55835, IRB-55671). The IRB approval of the study included a waiver of consent, on the basis that CDCR provided the Stanford research team with a

limited data set without direct identifiers, the data had been collected for operational purposes, and the study could not practicably be carried out otherwise. Similar approval conditions were met for California Department of Public Health data.

Sensitivity Analyses (SA)

We ran three sensitivity analyses to explore questions related to the fraction of co-workers unvaccinated and the history of Covid-19 variables, as follows. These were run on the custody staff group. Predicted probabilities and 95% confidence intervals are presented in Appendix Table 5.

SA 1: Within prison dispersion in the fraction of co-workers vaccinated

As noted in the text, our measure of co-worker peer influence is crude. To estimate the relationship between co-worker vaccination uptake and individual staff decision to remain unvaccinated, we would ideally see substantial variation across individual staff workers in the rate of vaccination of their co-worker cohorts within a given prison – a signal that there are differences in cohort-preferences for vaccination. If there is not substantial variation, it could be possible that exogenous shocks over time (not accounted for by the prison fixed effects) lead to heterogeneity in cohort vaccination rates. (Note: because the patterns of vaccination uptake are strikingly similar across prisons [see Appendix Figures 1 and 2] this concern about variation in the timing of vaccination due to such shocks is substantially reduced.) To explore this further, we examined the within-prison dispersion in the fraction of (custody staff) co-worker cohorts vaccinated and repeated our main regression analysis on the sample after excluding those prisons with the least variation. Within prisons, the mean fraction of unvaccinated custody workers was between 34% and 86%, the range (highest value – lowest value) was between 4 percentage points and 24 percentage points, and the interquartile range (IQR) between 1 and 8 percentage points. We re-ran our multivariable analysis limiting the sample to those prisons with a wider distribution of values (IQR ≥ median; 17 of 33 prisons included; N = 12,221) to examine whether individuals working in prisons with a wider dispersion were more or less likely to remain unvaccinated if their co-workers we less likely to be vaccinated. The results were robust to our main analysis, custody staff were six percentage points more likely to remain unvaccinated if their co-worker cohort was at the 75th percentile of being unvaccinated compared to working with a cohort at the 25th percentile (compared to being 5 percentage points more likely in our main analysis). See Appendix Table 5 for results.

SA 2: Interaction of main shift worked with fraction of co-workers vaccinated

It may be the case that peer interactions may be different for night shift versus day or swing shift and hence the relationship between co-worker vaccination patterns and staff remaining unvaccinated may be different by shift. To assess the possibility that main-shift-worked is an effect modifier (e.g., the correct model specification interacts the shift-variable with the fraction of co-worker vaccinated variable) in our model, we repeated the multivariable probit regression as in the main analysis expanded to include this interaction term. The coefficient on the interaction term was not significantly different than zero and the predicted probabilities were consistent with those resulting from our main specification. See Appendix Table 5 for results.

SA 3: History of Covid-19 by time of test

Because there is some concern that vaccination uptake is related to the timing of a previously positive SARS-CoV-2 test (e.g., an individual may be advised to wait 90 days from last positive test to receive a dose of vaccine), we divided our *History of Covid-19* variable into time periods as follows. The reference group (as in the main specification) is “no history of Covid-19” (N = 17,052), and the other categories are: last positive test prior to December 22, 2020 (N = 4,619); last positive test between December 22, 2020 and March 15, 2021 (N = 1,423); last positive test after March 15, 2021 (N = 378). We chose the time periods based on the idea that all those in the first and second groups (e.g., before March 15, 2021) would have time to become vaccinated within our study period even after a 90-day post infection window, and those in the first group would have time to be become vaccinated during the initial vaccine push evident in Figure 1 in the main text. We find that, for residents with last positive SARS-CoV-2 test prior to December 22, they are 5 percentage points more likely to remain unvaccinated compared to those with no prior history of Covid-19. For those with last positive SARS-CoV-2 test in the first few months of the study period, they are 13 percentage points more likely to remain unvaccinated. We can compare this to an estimate of those with prior infection in the main specification being 8-percentage points more likely to remain unvaccinated than those with no prior infection. Thus, when focusing on those with a history of prior Covid-19 more than 90 days before the end of our study period, history of prior Covid-19 remains a significant predictor of remaining unvaccinated.

© 2022 Prince L et al. *JAMA Health Forum.*

eTable 1: Counts of direct care staff, different zip codes of residence, and different counties of residence among CDCR custody and healthcare staff

	Custody staff			Healthcare Staff		
Prison	# Direct Care Staff	# of Unique Zip Codes of Residence	# of Counties of Residence	# Direct Care Staff	# of Unique Zip Codes of Residence	# of Unique Counties of Residence
1	675	72	9	96	32	8
2	358	75	8	64	17	4
3	687	37	6	97	30	8
4	502	81	30	59	11	10
5	921	63	11	151	40	8
6	524	69	12	213	50	13
7	656	35	6	101	31	7
8	1027	120	18	1518	177	31
9	805	179	8	267	112	12
10	457	147	8	289	121	6
11	799	42	13	271	34	11
12	822	115	20	641	125	28
13	1151	75	9	316	69	17
14	736	149	7	115	64	5
15	687	69	21	133	46	17
16	382	81	8	60	23	6
17	460	88	15	115	50	12
18	530	87	13	110	42	7
19	626	63	23	110	27	17
20	570	104	7	76	28	7
21	912	52	6	183	45	11
22	762	156	11	257	91	10
23	810	103	15	298	96	18
24	540	23	15	65	15	12
25	752	66	7	115	24	6
26	938	136	14	347	96	10
27	854	114	19	318	85	19
28	938	67	8	292	70	19
29	576	155	26	70	31	8

© 2022 Prince L et al. *JAMA Health Forum.*

30	666	106	21	137	54	11
31	991	254	35	256	105	15
32	868	127	26	299	101	23
33	490	63	9	178	44	10

All counts are derived from the analytic sample used in the multivariable analysis.

© 2022 Prince L et al. *JAMA Health Forum.*

eTable2: Fraction unvaccinated and fraction not vaccinated and without history of Covid-19 for prison for staff and incarcerated people; Cumulative incidence following the study period

	Incarcerated persons		Custody Staff		Healthcare staff		Cumulative Incidence (July 1 - Sept 25, 2021)
Prison	Not vaccinated no history of Covid-19 (%)	Not vaccinated (%)	Not vaccinated no history of Covid-19 (%)	Not vaccinated (%)	Not vaccinated no history of Covid-19 (%)	Not vaccinated (%)	Fraction of Incarcerated Persons (%)
1	6%	15%	39%	59%	39%	49%	0.12%
2	19%	36%	41%	71%	25%	38%	0.58%
3	22%	28%	31%	46%	46%	51%	0.03%
4	23%	41%	55%	76%	40%	50%	0.99%
5	19%	26%	49%	76%	43%	59%	0.31%
6	21%	28%	45%	60%	35%	47%	0.37%
7	17%	20%	25%	37%	38%	48%	0.11%
8	10%	13%	45%	59%	25%	30%	0.48%
9	13%	18%	32%	55%	26%	36%	0.21%
10	14%	21%	35%	50%	32%	38%	1.21%
11	8%	25%	35%	64%	23%	32%	0.26%
12	13%	19%	37%	48%	24%	28%	0.40%
13	18%	25%	44%	70%	37%	47%	0.49%
14	9%	22%	41%	59%	22%	36%	0.81%
15	6%	10%	40%	52%	38%	45%	0.04%
16	6%	16%	45%	61%	31%	36%	0.05%
17	13%	23%	48%	70%	22%	29%	0.00%
18	18%	30%	38%	53%	22%	31%	0.04%
19	14%	29%	45%	86%	48%	58%	0.58%
20	14%	28%	49%	66%	39%	52%	0.00%
21	30%	36%	46%	63%	35%	43%	0.03%
22	19%	30%	36%	60%	34%	43%	0.22%
23	7%	11%	48%	71%	38%	45%	0.46%
24	27%	31%	66%	82%	68%	79%	6.22%
25	20%	40%	48%	72%	51%	59%	0.94%
26	14%	19%	38%	58%	31%	37%	0.15%

© 2022 Prince L et al. *JAMA Health Forum.*

27	30%	34%	49%	65%	34%	39%	0.71%
28	13%	25%	43%	65%	45%	56%	0.32%
29	21%	37%	52%	70%	47%	54%	7.29%
30	21%	31%	42%	58%	31%	33%	3.38%
31	7%	16%	36%	42%	23%	26%	0.28%
32	31%	37%	36%	59%	26%	33%	0.00%
33	6%	13%	36%	51%	26%	34%	0.44%

Rates are based on our study sample (see main text for details). Cumulative incidence is the rate of new detected Covid-19 cases in incarcerated persons after the study period (from July 1, 2021 - September 25, 2021).

© 2022 Prince L et al. *JAMA Health Forum.*

eTable 3: Predicted probabilities and confidence intervals for adjusted analysis

	Custody staff		Healthcare staff	
	Probability	95% Confidence Interval	Probability	95% Confidence Interval
**Age**				
60+	0.45	(0.42 - 0.48)	0.29	(0.27 - 0.32)
50-59	0.51	(0.50 - 0.52)	0.31	(0.29 - 0.32)
40-49	0.57	(0.56 - 0.58)	0.36	(0.35 - 0.37)
30-39	0.68	(0.67 - 0.69)	0.44	(0.42 - 0.46)
18-29	0.75	(0.73 - 0.76)	0.52	(0.48 - 0.56)
**Racial/Ethnic Group**				
Asian/PI	0.53	(0.50 - 0.55)	0.3	(0.28 - 0.32)
Black	0.6	(0.58 - 0.62)	0.41	(0.38 - 0.44)
Hispanic	0.59	(0.59 - 0.60)	0.38	(0.37 - 0.40)
White	0.66	(0.64 - 0.67)	0.41	(0.38 - 0.43)
Other/Unknown	0.62	(0.60 - 0.63)	0.38	(0.36 - 0.41)
**Gender**				
Male	0.6	(0.60 - 0.60)	0.33	(0.31 - 0.35)
Female	0.67	(0.66 - 0.68)	0.39	(0.38 - 0.40)
**History of Covid-19 (before vaccination)**				
Yes	0.67	(0.66 - 0.68)	0.44	(0.42 - 0.47)
No	0.59	(0.59 - 0.60)	0.36	(0.36 - 0.36)
**Unvaccinated in Home Zip Code**				
75th%	0.63	(0.62 - 0.63)	0.4	(0.39 - 0.41)
Median	0.62	(0.61 - 0.62)	0.37	(0.37 - 0.37)
25th%	0.6	(0.59 - 0.60)	0.34	(0.33 - 0.35)
**Unvaccinated in Work Cohort**				
75th%	0.64	(0.62 - 0.66)	0.38	(0.36 - 0.41)
Median	0.61	(0.61 - 0.61)	0.36	(0.34 - 0.38)
25th%	0.59	(0.57 - 0.61)	0.35	(0.31 - 0.39)

See legend notes for Figure 3 in main text.

© 2022 Prince L et al. *JAMA Health Forum.*

eTable 4: Coefficients from multivariable analyses

	Custody staff	Healthcare staff
Age (compared to 20-29)		
30-39	-0.204***	-0.212***
40-49	-0.508***	-0.436***
50-59	-0.680***	-0.605***
60+	-0.827***	-0.653***
Race/Ethnicity (compared to Black)		
Asian/Pacific Islander	-0.215***	-0.334***
Hispanic	-0.028	-0.084*
White	0.147***	-0.018
Other/unknown	0.036	-0.093
Gender (Compared to Female)		
Male	-0.198***	-0.179***
History of Covid-19 (before vaccination)	0.230***	0.245***
Fraction of adults in zip code unvaccinated	0.669***	1.025***
Fraction of shift co-workers unvaccinated	0.904**	0.605
Main Shift worked (compared to night shift)		
Day shift	0.012	-0.083*
Swing shift	0.017	0.03
Number of shifts worked	-0.007***	-0.016***
Mean # shifts per week worked	0.127***	0.325***
Constant	0.138	-0.05
Observations	23,472	7,617
Prison Fixed Effects Included	Yes	Yes

*** p<0.01, ** p<0.05, * p<0.1

© 2022 Prince L et al. *JAMA Health Forum.*

	Main analysis (custody staff)		SA1: limited to prisons with more variation in fraction of co-workers not vaccinated		SA2: Interact shift most worked with unvaccinated in work cohort variable		SA3: History of Covid-19 by time period	
	Probability	95% Confidence Interval	Probability	95% Confidence Interval	Probability	95% Confidence Interval	Probability	95% Confidence Interval
**Age**								
60+	0.45	(0.42 · 0.48)	0.43	(0.39 · 0.47)	0.45	(0.42 · 0.48)	0.45	(0.42 · 0.48)
50-59	0.51	(0.50 · 0.52)	0.47	(0.45 · 0.49)	0.51	(0.50 · 0.52)	0.51	(0.50 · 0.52)
40-49	0.57	(0.56 · 0.58)	0.53	(0.52 · 0.55)	0.57	(0.56 · 0.58)	0.57	(0.56 · 0.58)
30-39	0.68	(0.67 · 0.69)	0.65	(0.64 · 0.66)	0.68	(0.67 · 0.69)	0.68	(0.67 · 0.69)
18-29	0.75	(0.73 · 0.76)	0.73	(0.71 · 0.76)	0.75	(0.73 · 0.76)	0.75	(0.73 · 0.76)
**Racial/Ethnic Group**								
Asian/PI	0.53	(0.50 · 0.55)	0.48	(0.44 · 0.51)	0.53	(0.50 · 0.55)	0.53	(0.50 · 0.55)
Black	0.60	(0.58 · 0.62)	0.59	(0.57 · 0.61)	0.60	(0.58 · 0.62)	0.60	(0.58 · 0.62)
Hispanic	0.59	(0.59 · 0.60)	0.57	(0.55 · 0.58)	0.59	(0.59 · 0.60)	0.59	(0.58 · 0.60)
White	0.66	(0.64 · 0.67)	0.62	(0.61 · 0.64)	0.65	(0.64 · 0.67)	0.65	(0.64 · 0.67)
Other/Unknown	0.62	(0.60 · 0.63)	0.58	(0.57 · 0.60)	0.62	(0.60 · 0.63)	0.62	(0.60 · 0.63)
**Gender**								
Male	0.60	(0.60 · 0.60)	0.57	(0.57 · 0.58)	0.60	(0.60 · 0.60)	0.60	(0.60 · 0.60)
Female	0.67	(0.66 · 0.68)	0.62	(0.60 · 0.64)	0.67	(0.66 · 0.68)	0.67	(0.66 · 0.68)
**History of Covid-19 (before vaccination)**								
Yes	0.67	(0.66 · 0.68)	0.63	(0.61 · 0.66)	0.67	(0.66 · 0.68)		
No	0.59	(0.59 · 0.60)	0.56	(0.55 · 0.57)	0.59	(0.59 · 0.60)		
**History of Covid-19, by date of last positive test before vaccination**								
No history							0.59	(0.59 · 0.60)
Before Dec 22, 2020							0.64	(0.63 · 0.65)
Dec 22, 2020 to March 15, 2021							0.72	(0.70 · 0.75)
After March 15, 2021							0.85	(0.80 · 0.89)
**Unvaccinated in Home Zip Code**								
75th%	0.63	(0.62 · 0.63)	0.60	(0.59 · 0.61)	0.63	(0.62 · 0.63)	0.63	(0.62 · 0.63)
Median	0.62	(0.61 · 0.62)	0.58	(0.58 · 0.59)	0.62	(0.61 · 0.62)	0.62	(0.61 · 0.62)
25th%	0.60	(0.59 · 0.60)	0.57	(0.56 · 0.57)	0.60	(0.59 · 0.60)	0.60	(0.59 · 0.60)
**Unvaccinated in Work Cohort**								
75th%	0.64	(0.62 · 0.66)	0.61	(0.59 · 0.64)	0.64	(0.62 · 0.66)	0.64	(0.62 · 0.66)
Median	0.61	(0.61 · 0.61)	0.59	(0.58 · 0.59)	0.61	(0.61 · 0.61)	0.61	(0.61 · 0.61)
25th%	0.59	(0.57 · 0.61)	0.55	(0.54 · 0.57)	0.59	(0.57 · 0.61)	0.59	(0.57 · 0.61)

See legend notes for Figure 3 in main text.

© 2022 Prince L et al. *JAMA Health Forum.*

eFigure 1: Variation among prisons in vaccine and test-positive scale-up over time, Custody staff

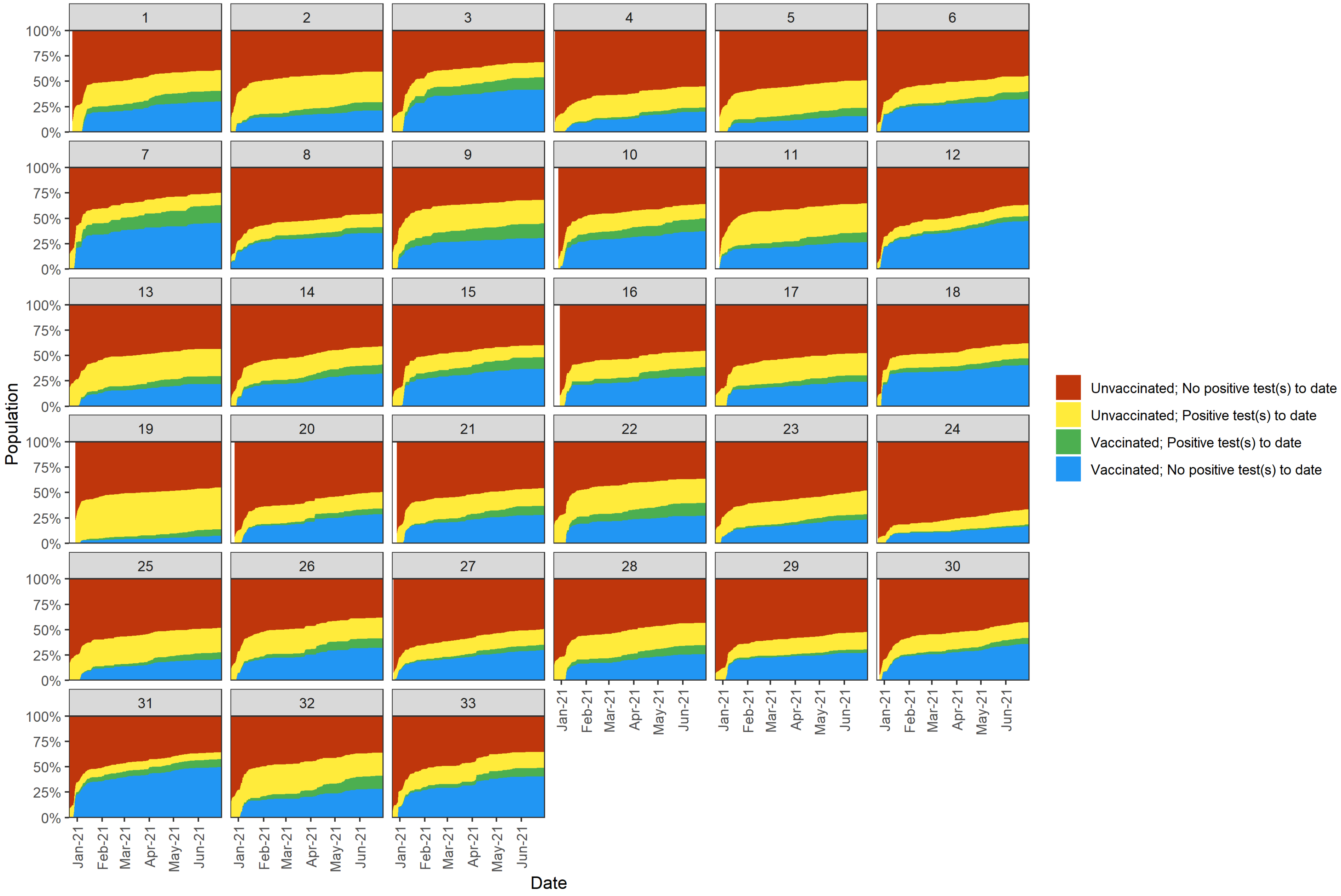

© 2022 Prince L et al. *JAMA Health Forum.*

eFigure 2: Variation among prisons in vaccine and test-positive scale-up over time Healthcare staff

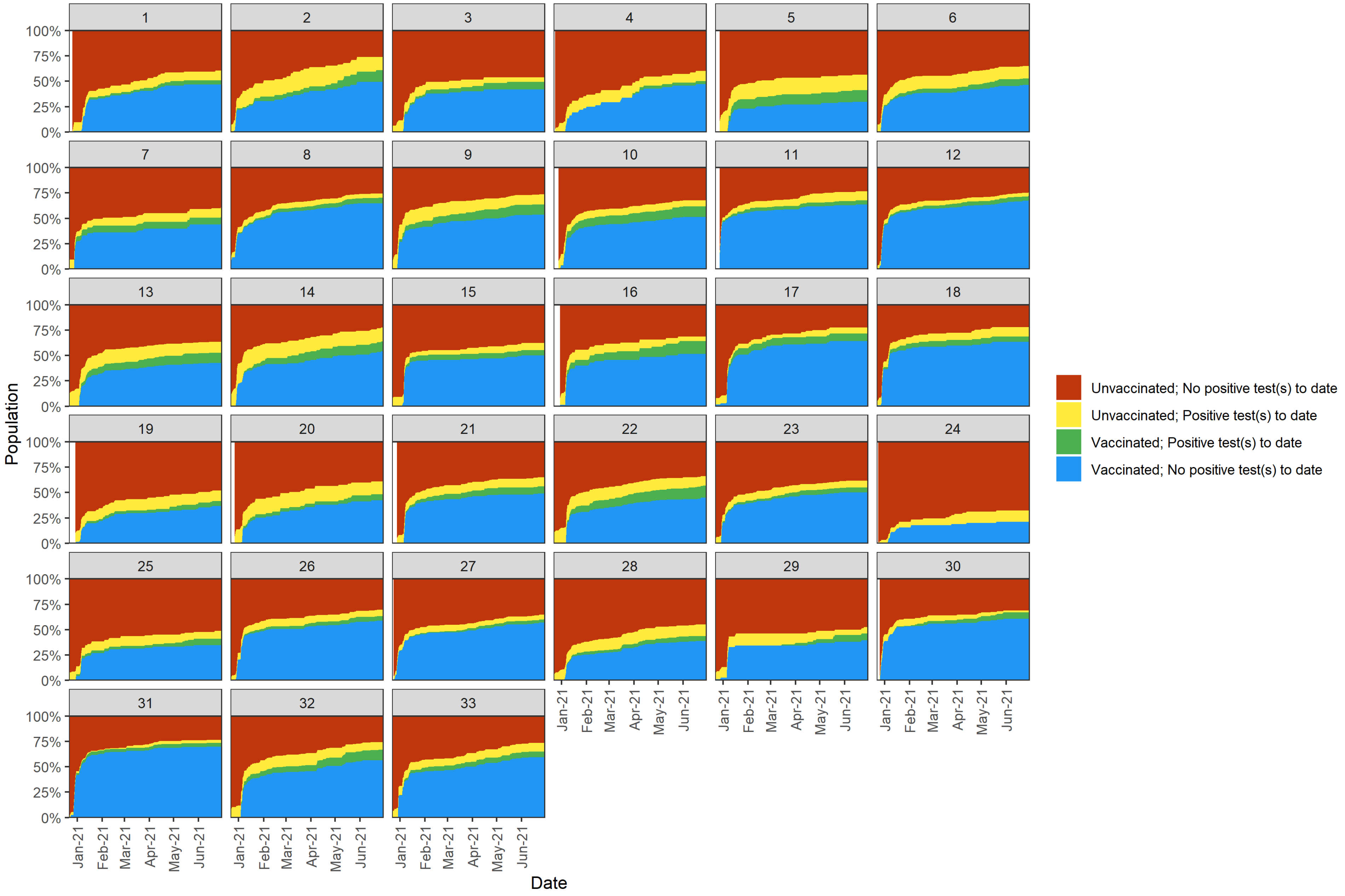

© 2022 Prince L et al. *JAMA Health Forum.*

eFigure 3: Counts of COVID-19 deaths among staff and fraction of direct care staff vaccinated, by institution (December 2020 – June 30, 2021)

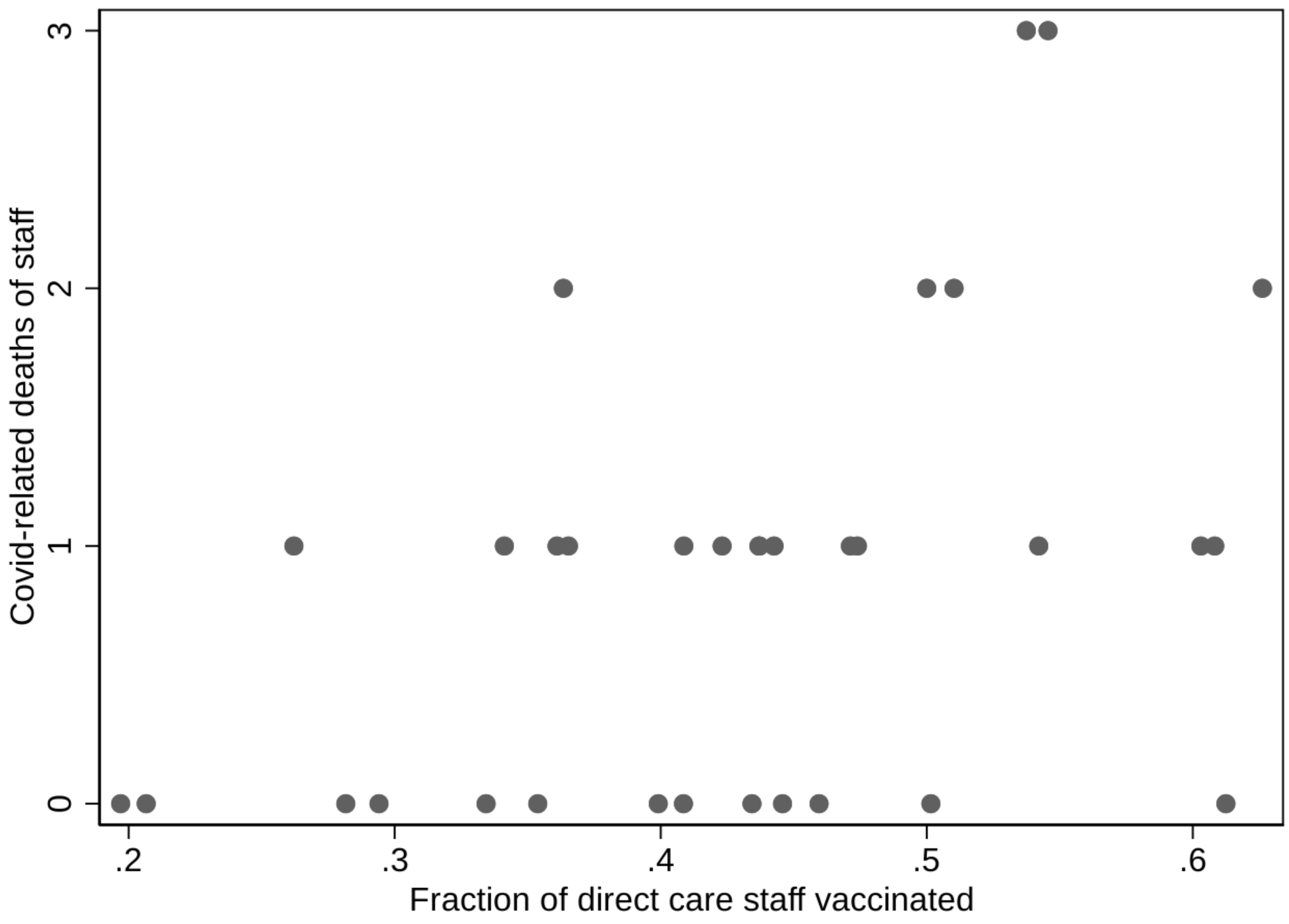

Scatter points for each of the 33 institutions included in the analysis. Fraction of direct care staff vaccinated is the total number of custody and healthcare staff vaccinated divided by the total number of staff included in the analyses (December 2020 – June 31, 2021). Covid-related deaths of staff are from the website https://www.cdcr.ca.gov/covid19/cdcr-cchcs-covid-19-status/ as of January 10, 2022. In a test of correlation between fraction vaccinated and covid-related deaths of staff, the Spearman's rho = 0.4484.

© 2022 Prince L et al. *JAMA Health Forum.*
